# m6ATM: a deep learning framework for demystifying the m6A epitranscriptome with Nanopore long-read RNA-seq data

**DOI:** 10.1093/bib/bbae529

**Published:** 2024-10-22

**Authors:** Boyi Yu, Genta Nagae, Yutaka Midorikawa, Kenji Tatsuno, Bhaskar Dasgupta, Hiroyuki Aburatani, Hiroki Ueda

**Affiliations:** Advanced Data Science Division, Research Center of Advanced Science and Technology, The University of Tokyo, 4-6-1 Komaba, Meguro-ku 153-8904, Tokyo, Japan; Genome Science & Medicine Division, Research Center of Advanced Science and Technology, The University of Tokyo, 4-6-1 Komaba, Meguro-ku 153-8904, Tokyo, Japan; Department of Digestive Surgery, Nihon University School of Medicine, 30-1 Oyaguchi Kami-cho, Itabashi-ku 173-8601, Tokyo, Japan; Genome Science & Medicine Division, Research Center of Advanced Science and Technology, The University of Tokyo, 4-6-1 Komaba, Meguro-ku 153-8904, Tokyo, Japan; Advanced Data Science Division, Research Center of Advanced Science and Technology, The University of Tokyo, 4-6-1 Komaba, Meguro-ku 153-8904, Tokyo, Japan; Genome Science & Medicine Division, Research Center of Advanced Science and Technology, The University of Tokyo, 4-6-1 Komaba, Meguro-ku 153-8904, Tokyo, Japan; Advanced Data Science Division, Research Center of Advanced Science and Technology, The University of Tokyo, 4-6-1 Komaba, Meguro-ku 153-8904, Tokyo, Japan

**Keywords:** epitranscriptomics, deep neural networks, long-read RNA sequencing, m6A

## Abstract

N^6^-methyladenosine (m6A) is one of the most abundant and well-known modifications in messenger RNAs since its discovery in the 1970s. Recent studies have demonstrated that m6A is involved in various biological processes, such as alternative splicing and RNA degradation, playing an important role in a variety of diseases. To better understand the role of m6A, transcriptome-wide m6A profiling data are indispensable. In recent years, the Oxford Nanopore Technology Direct RNA Sequencing (DRS) platform has shown promise for RNA modification detection based on current disruptions measured in transcripts. However, decoding current intensity data into modification profiles remains a challenging task. Here, we introduce the m6A Transcriptome-wide Mapper (m6ATM), a novel Python-based computational pipeline that applies deep neural networks to predict m6A sites at a single-base resolution using DRS data. The m6ATM model architecture incorporates a WaveNet encoder and a dual-stream multiple-instance learning model to extract features from specific target sites and characterize the m6A epitranscriptome. For validation, m6ATM achieved an accuracy of 80% to 98% across *in vitro* transcription datasets containing varying m6A modification ratios and outperformed other tools in benchmarking with human cell line data. Moreover, we demonstrated the versatility of m6ATM in providing reliable stoichiometric information and used it to pinpoint PEG10 as a potential m6A target transcript in liver cancer cells. In conclusion, m6ATM is a high-performance m6A detection tool, and our results pave the way for future advancements in epitranscriptomic research.

## Introduction

RNA modifications have gained prominence as key targets in biological processes [1–3]. Among over 150 types of modifications, N^6^-methyladenosine (m6A) stands out in recent research due to its remarkable prevalence and profound functional significance [4]. In mammalian messenger RNAs (mRNAs), evidence suggests that m6A modifications are preferentially installed in DRACH motifs (where D = G/A/U, R = G/A, H = A/U/C) [5, 6]. It has been reported that m6A governs the fate of RNA via diverse mechanisms such as RNA translation, degradation, and alternative splicing. Not surprisingly, recent research has demonstrated that dysregulation of m6A contributes to a wide range of diseases, including neurological disorders, cardiovascular diseases, and cancers [7, 8]. Consequently, it is of great significance to gain a deeper understanding of m6A based on its transcriptome-wide landscape.

One challenge in transcriptome-wide m6A detection is selecting the appropriate method for different studies. To date, there is no gold-standard method for transcriptome-wide m6A detection. Conventionally, short-read RNA sequencing (RNA-seq) technologies have been widely used for identifying m6A sites. However, these methods come with limitations, including low resolution, reagent-based biases, and tedious preparation procedures. For instance, MeRIP-seq [9], a frequently used traditional m6A profiling method, was first proposed in 2012. This method uses anti-m6A antibodies to selectively enrich RNA fragments marked with m6A for high-throughput sequencing, but its main limitation is the low resolution of $\sim $100 nucleotides. Methods developed later, such as miCLIP-seq [6] in 2015, MAZTER-seq [10] in 2019, m6A-SAC-seq in 2022 [5], and GLORI-seq [11] in 2023, have enhanced the resolution to single-nucleotide levels, providing a more detailed view of the m6A landscape. However, a major limitation of these methods is the reagent-induced bias, which may arise from nonspecific antibody binding or preferential enzyme targeting. These unwanted detection biases can lead to inaccurate predictions of m6A sites and unreliable estimations of m6A stoichiometry.

In recent years, the Nanopore Direct RNA Sequencing (DRS) platform has emerged as a promising choice for RNA modification detection due to its ability to directly sequence RNA molecules in real time while preserving modification information [12]. Specifically, the DRS platform uses discriminative patterns of ionic currents generated during the translocation of a single RNA molecule through a nanopore protein to determine its nucleic acid sequences. One pioneering study [13] highlighted the mean current amplitude difference between m6A-modified and unmodified GGACU motifs, opening new avenues for developing m6A detection strategies. Unlike short-read profiling methods, the DRS library preparation does not require additional treatments or amplification, producing results free from reagent- and amplification-induced biases. Furthermore, the ability to sequence full-length transcripts enables it to resolve complex isoforms, which is challenging for short-read data. However, using DRS data for m6A detection is challenging due to the interpretation of ambiguous ionic current patterns. Noisy signals from homopolymers and secondary structures can lead to high error rates in basecalled sequences. Moreover, previous studies have noted that m6A modifications in specific 5-mer motifs produce limited current changes[14, 15]. Consequently, a sophisticated computational tool is required for accurately decoding the inherent information in ionic current data.

Various computational approaches based on signatures derived from ionic currents have been applied to DRS data for m6A detection [16, 17]. A groundbreaking tool, EpiNano [18], harnesses systematic basecalling “errors” such as deletions, mismatches, and base qualities. It employs machine learning models or pairwise comparisons to interpret errors as modification profiles. Other early-developed methods, such as Tombo [19] and MINES [14], use re-annotated current intensity data for noncanonical base prediction. Specifically, Tombo first resquiggles raw current intensity data to reference sequences and identifies discriminative signal patterns different from canonical nucleotides using statistical models, while MINES applies a random forest model to modification fraction values estimated by Tombo for m6A prediction. However, the accuracy of these early approaches was not very promising.

Recent supervised methods such as m6Anet [20] and m6Abasecaller [21] have leveraged deep neural networks to achieve more accurate predictions. m6Anet is a site-level m6A detection tool that applies a multiple-instance learning (MIL) model to learn from both current intensity and sequence features of the 5-mer sequence context, while m6ABasecaller is a molecular-level detection tool that incorporates an m6A basecalling model to predict m6A in addition to the four canonical bases (A, C, G, T, and m6A) within the Guppy basecaller. However, m6Anet uses a small and simple neural network that takes summary statistics of the ionic current data as input, potentially obscuring valuable information from the current patterns. Additionally, m6Anet requires a signal segmentation tool, Nanopolish, for preprocessing, which adds extra complexity. On the other hand, m6ABasecaller directly recognizes m6A modifications through a basecalling algorithm. While this method theoretically enables detection at the molecular level, detecting modifications without realigning signals to a reference in single reads remains a considerable challenge, requiring further detailed validation and comparison. Despite these limitations, the improved performance of these two methods compared with early approaches demonstrates the potential of deep neural networks for m6A prediction. Similar to approaches used in Nanopore basecallers and speech recognition [22], detection accuracy can be improved by learning the patterns of the current signal itself. Thus, it is crucial to consider a more effective deep learning approach to capture valuable information from voluminous sequential data.

Convolutional neural networks (CNNs) [23, 24] have been well known for their breakthroughs in image recognition and object detection tasks. Additionally, CNNs have achieved remarkable success beyond image-related applications, such as speech recognition and electrocardiogram monitoring, using one-dimensional variants known as 1D CNNs [25]. DeepBinner [26], for instance, applied 1D CNNs to identify both local patterns and temporal dependencies within Nanopore signal data for DNA barcode demultiplexing. Their results supported the use of 1D CNNs for processing time-series Nanopore sequencing data, wherein each observation was influenced by past observations.

**Figure 1:**
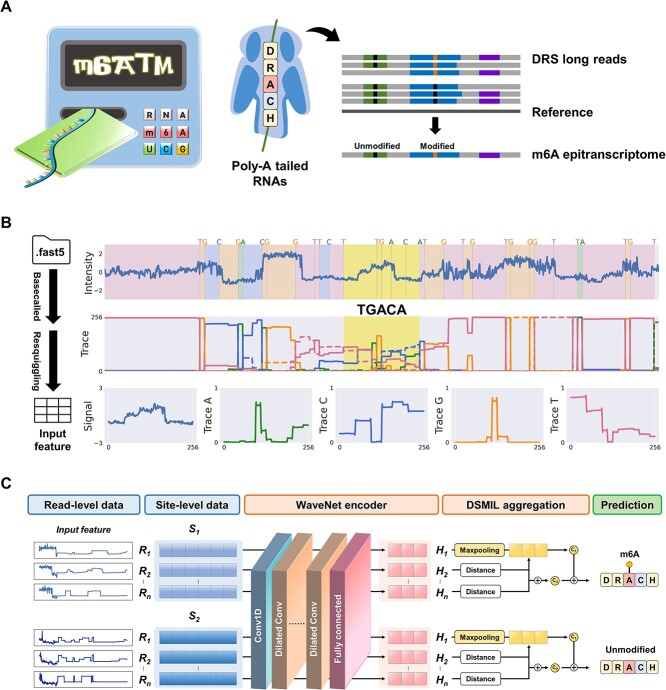
Schematic diagram of m6ATM. (A) m6ATM was designed to predict m6A modifications at a given DRACH site in the reference transcriptome using DRS data. (B) Data preprocessing module for m6ATM. Raw FAST5 files were base-called, aligned, and resquiggled to generate read-level features containing signal and trace data. The yellow block represents the target interval of the five channels (signal and trace A/C/G/T) at the TGACA query site. The signal and trace values were transformed and concatenated into the final read-level feature of length 1280. (C) Architecture of WaveNet-DSMIL model for m6A prediction. The model aggregated 20–1000 reads at each site to determine whether the site is m6A-modified. (R: read-level data, S: site-level data, H: features from hidden layers).

In this study, we propose m6A Transcriptome-wide Mapper (m6ATM) as a robust solution for transcriptome-wide m6A profiling (Fig. 1). To preserve the inherent nature of m6A-modified nucleotides in ionic currents, we implemented dilated 1D convolutional layers from a deep generative model of audio waveforms, WaveNet [27], as a potent approach for encoding distinctive read-level representations. Additionally, a dual-stream architectural neural network was applied to translate read-level representations into site-level m6A predictions. We showed that m6ATM is exceptionally proficient at extracting features from raw DRS data, and its effectiveness was confirmed through testing with synthetic *in vitro* transcription (IVT) sequences and human cell line data. Overall, our results suggest that m6ATM is a high-performance computational method for m6A detection, and its Python package is available at https://github.com/poigit/m6ATM.

## Materials and methods

### Public datasets

Raw FAST5 files of IVT datasets containing 100% m6A-modified and unmodified adenosines were retrieved from the NCBI Gene Expression Omnibus repository under accession number GSE124309 [18]. Based on the original article’s description, the synthetic IVT sequences, $\sim $2000 nucleotides in length, were designed using CURLCAKE (http://cb.csail.mit.edu/cb/curlcake/) to include all possible combinations of 5-mer nucleotides and minimize the effect of RNA secondary structures. Raw FAST5 files of the human cell lines HEK293 and HEK293T were retrieved from the NCBI Gene Expression Omnibus repository under accession number GSE132971 [14] and the European Nucleotide Archive under accession number PRJEB40872 [28], respectively.

### Data retrieval and normalization

Raw FAST5 files containing current intensity data were basecalled by Guppy basecalling software version 5.0.11 to generate FASTQ files of the basecalled sequences. Reads with a quality score higher than seven were used for alignment. Sequence alignment was performed against synthetic IVT sequences or the GRCh38 reference transcriptome (cDNA and ncRNA) retrieved from the Ensembl project using Minimap2, and sorted BAM files and index files were prepared using Samtools. Codes for data preprocessing are available at https://github.com/poigit/m6ATM.

The read-level raw current intensity data (signal) and base probability data (trace) provided by Guppy were collected in intermediate HDF5 files. To remove unwanted variations in the signal data, a median-based modified Z-score was used to standardize the current intensity values as follows:


\begin{align*} & Modified\ Z\ score = \frac{0.6745(x_{i} - \tilde{x})}{MAD}, \end{align*}

where $x_{i}$ is a single sampling value of the signal data, $\tilde{x}$ is the median of all sampling values, $MAD$ is the median absolute deviation, and 0.6745 is the 75th percentile of the normal distribution used for consistency.

Trace data, which are generated by the recurrent neural network of Guppy, store probability values ranging from 0 to 255 for eight types of bases: the four standard nucleotides (A, C, G, and U) and four polynucleotides (AA, CC, GG, and UU). Specifically, the value of trace data is derived from chunks of raw current intensity data, where every 10 data points are condensed into a single data point in trace data. These probabilities were integrated into the four standard bases and scaled to values between 0 and 1. The normalization process of the trace data is summarized as follows:


\begin{align*} & T = (p_{A}, p_{C}, p_{G}, p_{U}, p_{AA}, p_{CC}, p_{GG}, p_{UU}); \end{align*}

\begin{align*} & q_{i} =p_{i} + p_{ii}; \end{align*}

\begin{align*} & T_{Normalized} = (q_{A}, q_{C}, q_{G}, q_{U}), \end{align*}

where $T$ denotes the trace data, $p$ is the probability from 0 to 255, $q$ is the normalized value from 0 to 1, and $i$ represents nucleotides A, C, G, and T.

### Trace-based resquiggling

To collect features from a target site, signal/trace segmentations based on reference sequences were required. Thus, we applied the trace-based Viterbi algorithm for resquiggling [29]. First, predicted basecalling breaks in the CTC encoder of the Guppy basecaller were mapped to trace data based on the “move” values. A change point detection algorithm was then used to split data intervals longer than 1.5 times the median length at the midpoint and add potential segmentation boundaries. Trace values were further aggregated from eight types into the standard four bases per segmentation boundary, and the Smith–Waterman algorithm was applied to realign these trace values with the reference sequence contexts. We used a dynamic programming approach to improve trace value alignment on the reference and calculated the alignment score $A$ as follows:


\begin{align*} & A = W(S_{match} - S_{mismatch}) \end{align*}

Here, $S_{match}$ is the score for correctly matched signal values, and $S_{mismatch}$ is the score for mismatches, based on how well the trace values align with the reference. The weight $W$, which is an adjustment factor based on the average length of the segments, was adjusted to 1 for simplicity. Next, we calculated the transition probability $P_{trans}$ as


\begin{align*} & P_{trans} = dot\ product(Trace\ value[n][m],\ Reference base) \end{align*}

We then filled a matrix with scores as follows:


\begin{align*} & S_{trans} = A \cdot P_{trans}; \end{align*}

\begin{align*} & S_{non-trans} = A \cdot (1-P_{trans}), \end{align*}

where $S_{trans}$ is the score for moving to the next base and $S_{non-trans}$ is the score for not moving to the next base. The final score $S$ at each point can be represented as


\begin{align*} & S = max(S_{trans},\ S_{non-trans}) \end{align*}

With $n$ and $m$ marking positions in the score matrix, we start from the end and work backward through the data using the traceback algorithm to determine the most likely series of trace signal realignments.

### Data collection and transformation

Both signal and trace data were retrieved from a 5-nt sequence with a central adenosine and 2-nt flanking windows, considering only DRACH sites. To standardize the lengths of both signal and trace data, we applied a multiplication factor of 10 to trace data. The variable data length between different reads was standardized to a length of 256 using interpolation or downsampling strategies.

### Read-level feature encoder

To generate robust read-level representations, we incorporated a WaveNet [27] encoder into our model architecture. The encoder comprises a stack of dilated causal convolutional layers to capture long-term dependencies, and a post-processing block to reduce computational costs while retaining essential information. Dilations enabled the network to capture large receptive fields. We modified WaveNet to convert each read-level input data at a given site into its feature representations. The modified architecture is a lightweight WaveNet encoder with two residual blocks, each containing three dilated convolutional layers with dilation factors of 1, 2, and 4. Skip connections from all residual blocks were summed and fed into the convolutional and fully connected layers, generating an embedding vector with a dimensionality of 1024. Details of the WaveNet architecture are shown in Figure S2.

### Site-level m6A prediction model

For m6A prediction, we applied a modified Dual-Stream Multiple Instance Learning (DSMIL) model [30] to convert “read-level” features into “site-level” information. MIL is a machine learning technique used for classification tasks where the input data are represented as a collection of unlabeled “instances” [31, 32]. MIL classifies the entire collection, commonly referred to as a “bag,” based on the bag-level label without prior knowledge of individual instances. The DSMIL model has demonstrated high accuracy in translating “pixel-level” instances into “slide-level” labels in whole slide image classification tasks. We adapted this model for m6A prediction by modifying the MIL aggregator to accept the WaveNet-encoded features. Specifically, the first stream of the aggregator uses a max-pooling layer on the read-level features $H_{i}$ at site $S$ to derive the score $C_{1}$ of the critical instance:


\begin{align*} & S = \{H_{1}, H_{2},..., H_{n}\} \end{align*}

\begin{align*} & C_{1} = Maxpool(S) \end{align*}

In the second stream, the network aggregates all the read-level features into a site-level embedding $B$, considering the proximity of other instances to the critical instance $H_{m}$:


\begin{align*} & v_{i} = W_{v}H_{i}; \end{align*}

\begin{align*} & B = \sum_{i}^{n-1} U(H_{i}, H_{m})v_{i}, \end{align*}

where $v_{i}$ is an information vector derived from feature $H$ based on weight vector $W_{v}$, and $U$ is the distance measurement function proposed in the original publication of DSMIL [30]. The final site-level indicator for m6A prediction was obtained by averaging the weighted score $C_{2}$ derived from $B$ with the score $C_{1}$. The probability of modification is defined as


\begin{align*} & C_{2} = W_{b}B; \end{align*}

\begin{align*} & C_{1} = \frac{1}{2}(C_{1} + C_{2}); \end{align*}

\begin{align*} & P(mod) = softmax(C), \end{align*}

where $W_{b}$ is the weight vector for $C_{2}$ and $P(mod)$ is the probability of m6A modification, ranging from 0 to 1.

### Stoichiometric estimation

To estimate the m6A stoichiometry at each site, we used a separate neural network consisting of fully connected layers that transform the read-level feature $H$ into pseudo-labels of 0 or 1. The m6A modification ratio is defined as the average value of these pseudo-labels. Details of the neural network are provided in Figure S5.

### Metagene analysis

The transcriptomic coordinates of m6A sites were converted into genomic coordinates based on the GRCh38 reference genome. We calculated the density of m6A peaks across genomic regions (3’UTR, CDS, 5’UTR) using the R package Guitar v2.14.0 [33].

### Silhouette analysis

Silhouette analysis [34] was used to evaluate the performance of clustering methods. For each object, the silhouette score was calculated as follows:


\begin{align*} & s(obj) = \frac{b(obj) - a(obj)}{max\{a(obj), b(obj)\}}, \end{align*}

where $a$ is the average distance between the query object and all objects in the same cluster, and $b$ is the average distance between the query object and objects in other clusters. The scores range from -1 to 1, with a score close to 1 indicating well-clustered objects, a score of 0 indicating overlapping clusters, and a score of -1 indicating inappropriate clustering. The average silhouette score is used to interpret the clustering results.

### eCLIP data analysis

We retrieved preprocessed HepG2 ENCODE eCLIP data from the POSTAR3 database [35, 36]. Fisher exact test was used to examine the enrichment of eCLIP peaks from 103 RNA binding proteins (RBPs) datasets in the predicted m6A peaks. Datasets without any overlap with DRACH sites in defined gene regions were excluded from analysis. The odds ratio for enriched eCLIP peaks at m6A-modified DRACH sites over unmodified DRACH sites was calculated using the unconditional maximum likelihood estimate via the Python package SciPy [37]. Results were considered significant when the *P* value was ¡0.05.

### Short-read RNA-seq data analysis

Short-read RNA-seq data of hepatocellular carcinoma (HCC) patients used in this study were sourced from our previous study [38] and The Cancer Genome Atlas (TCGA) database [39]. We retrieved the TCGA-LIHC (Liver Hepatocellular Carcinoma) dataset using the R package TCGAbiolinks [39, 40]. Our HCC RNA-seq data consist of 160 tumor samples and 31 adjacent normal tissue samples, while the TCGA-LIHC (Liver Hepatocellular Carcinoma) data include 371 primary solid tumor samples and 50 normal tissue samples. Gene expression values were log2 transformed fragments per kilobase of transcript per million mapped reads (FPKM). To compare gene expression differences among HCC tumors with varying PEG10 levels, samples were categorized into three groups based on their PEG10 expression levels. The “PEG10-High” group includes samples with expression levels at or above the third quartile (Q3), while the “PEG10-Low” group consists of samples with expression levels below the first quartile (Q1). The remaining samples were assigned to the “PEG10-Mid” group. The Wilcoxon signed-rank test was used to evaluate the differences between the PEG10-High and PEG10-Low group, and all results were considered significant when the *P* value was ¡0.05. Heatmap visualization was performed using the R package pheatmap [41].

## Results

### Comprehensive m6A modification analysis using m6ATM

We developed a novel computational tool, m6ATM, based on deep neural networks to detect transcriptome-wide, site-level m6A modifications (Fig. 1A). Briefly, m6ATM uses a collection of reads to characterize each transcriptomic site and determine whether it is m6A-modified or not. The computational pipeline includes three key steps: data preprocessing, feature encoding, and prediction. The data preprocessing module resquiggles current intensity data in FAST5 format against a reference transcriptome, collecting read-level features including signal (current intensity) and trace (base probability) for each DRACH site (Fig. 1B). Trace data, referring to a time series of probability values, are generated by the recurrent neural network of the Nanopore Guppy basecaller, recording continuous changes in currents and reflecting the sequence of nucleotides passing through the nanopore. Theoretically, trace data provide refined, denoised information compared with signal data, complementing the details from the signal data. Existing DRS-based m6A detection tools primarily rely on external resquiggling software, such as Nanopolish or Tombo [19], which requires additional execution steps and adds complexity for users. We integrated the Viterbi-based resquiggling algorithm from our previous study into our m6A detection tool, creating a user-friendly environment for data preprocessing. To predict site-level m6A modifications, we adapted the previously proposed DSMIL model to process read representations, which were converted from signal and trace values using a WaveNet encoder (Figs. 1B and 1C). For each DRACH site, 20–1000 read representations were aggregated to create a site-level representation. Finally, the site-level representation was used to infer the probability of m6A modification in the DSMIL prediction model.

### m6ATM accurately predicts m6A sites by WaveNet-DSMIL

**Figure 2:**
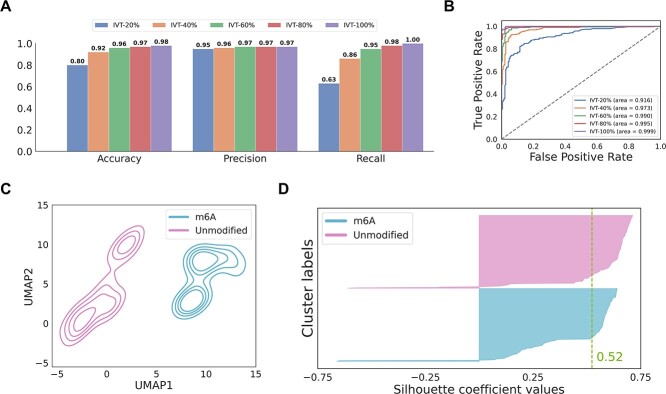
The model performance of m6ATM. (A) Accuracy, precision, and recall values of m6A prediction results in *in silico* mixed IVT datasets with different modification ratios (20%, 40%, 60%, 80%, and 100%). A probability of $\ge $0.5 was set as the threshold for determining a positive prediction. (B) ROC curves of m6A modification prediction results in *in silico* mixed IVT datasets. (C) The contour plot of UMAP transformed site-level features at 191 DRACH sites using both signal and trace data. (D) The silhouette plot of UMAP clusters in (C), indicating the similarity of clusters. The green vertical line in the silhouette plot represents the average silhouette coefficient (1 for well-separated clusters, 0 for overlapping clusters).

To train and validate the model, we used two biological replicates of IVT datasets to generate *in silico* mixed m6A positive/negative sites with 20%–100% modified reads and 0% modified reads, respectively (Figure S3). The results indicated that the model achieved an accuracy of over 92% and an area under the receiver operating characteristic curve (ROC-AUC) of over 0.973 in validation datasets with no less than 40% modified reads (Figs. 2A and 2B). Notably, even in the dataset containing a low modification ratio of 20%, our m6A prediction model exhibited commendable performance with 80% accuracy and an ROC-AUC score of 0.916.

Given that the role of trace data in predicting m6A modifications is not well understood, we investigated whether using a combination of signal and trace data could improve model performance. We compared site-level features from 382 IVT sites, including both m6A-modified and unmodified sites, under two feature extraction strategies: one combining signal and trace data and the other using only signal data (Fig. 2C and Figure S4). Uniform Manifold Approximation and Projection (UMAP) clustering analysis revealed distinct separation between 100% m6A-modified and unmodified sites when using both signal and trace data, as indicated by a silhouette coefficient of 0.52. This distinct separation was not observed when only signal data were used, as the silhouette coefficient dropped to 0.05 (Figure S4), suggesting that trace data preserve discriminative signatures effective for m6A detection. Additionally, as the modification ratio decreased to 60% in the group using both signal and trace data, the m6A-modified cluster moved closer to the unmodified cluster in the UMAP analysis. This shift was even more evident with 20% m6A modification, where overlap between the two clusters was reflected by a decrease in the silhouette coefficient to 0.04. This finding corresponds with the performance evaluation in Fig. 2A, where a decline in accuracy to 0.8 was noted in the 20% m6A modification group.

### Comparison of other m6A detection methods

**Figure 3:**
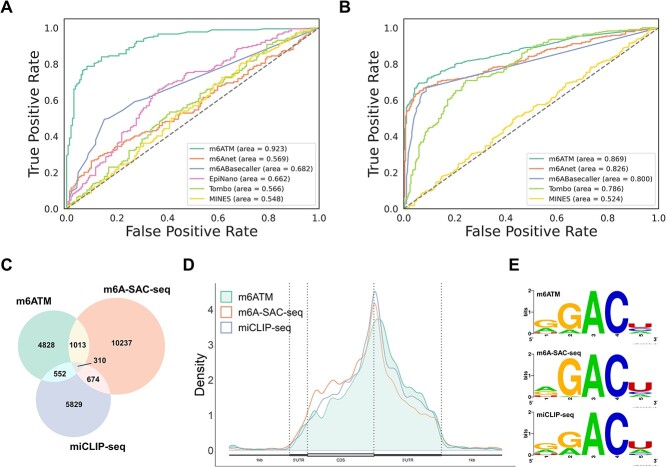
Comparison of DRS-based and short-read-based methods. (A) ROC curves for m6A prediction of *in silico* mixed IVT data using DRS-based tools. AUC scores are shown in the figure legend. Positive IVT data consist of 20% m6A-modified reads, while negative IVT data contain no modified reads. For fair comparison, only sites that can be detected by all methods were used. All 348 sites, including 174 positive and 174 negative, were used for validation. (B) ROC curves for m6A prediction in HEK293 cells using DRS-based tools. Only sites meeting the following two conditions were included: (1) at least 50 reads coverage, (2) detectable by all methods. All 570 sites were used for validation, including 285 positive sites identified from previously reported m6A sites in m6A-SAC-seq data, and 285 negative sites randomly sampled from sites not reported as m6A-modified in previous methods including m6A-SAC-seq and miCLIP-seq. EpiNano was excluded from the analysis because it could not complete the job within two weeks. (C) Venn diagram of HEK293 m6A sites identified by three detection methods: m6ATM, miCLIP-seq, and m6A-SAC-seq. A probability of $\ge $0.9 was set as the threshold for determining a positive prediction with m6ATM. (D) Metagene plot showing the distribution of HEK293 m6A positive sites across genomic locations. (E) Motif frequency of HEK293 m6A sites identified by the three detection methods.

To rigorously evaluate our model’s performance, we benchmarked m6ATM against previously published DRS-based computational tools: Tombo, EpiNano, MINES, m6Anet, and m6ABasecaller. First, we used the *in silico* mixed 20% IVT data to evaluate model performance under low-modification conditions (Fig. 3A). Our tool, m6ATM, demonstrated improved performance compared with other m6A detection tools (ROC-AUC = 0.923), underscoring its advantages, as previous studies have shown that m6A modification frequencies are typically low at most sites in cells. Additionally, we used human HEK293 cell line data to assess the effectiveness of each computational tool based on 285 m6A sites reported by m6A-SAC-seq and 285 DRACH sites randomly sampled from sites not reported as m6A-modified in short-read-based methods including m6A-SAC-seq and miCLIP-seq (Fig. 3B and Figure S6). In terms of ROC-AUC and area under the precision-recall curve (PR-AUC), m6ATM emerged as a leading benchmarking method (ROC-AUC = 0.869 and PR-AUC = 0.9). To eliminate potential bias from randomness, we repeated the analysis 10 times for confirmation (Figure S6B). Notably, m6ATM consistently outperformed other methods, achieving an average AUC-ROC of 0.854 and an average PR-ROC of 0.877. Overall, all tools scored above 0.7, except MINES, in the benchmark test. The low MINES score may be due to its initial design for only four DRACH motifs; however, we expanded its evaluation to include all 18 DRACH motifs to ensure fairness in comparison.

To evaluate the concordance of our prediction results with other short-read-based m6A sequencing methods at single-nucleotide resolution, we used m6ATM to identify m6A sites in HEK293 cells and compared the findings with those of two previously published approaches: m6A-SAC-seq and miCLIP-seq. The Venn diagram showed that m6ATM could identify novel m6A-positive sites (n = 4828) not detected by the other methods (Fig. 3C). However, only a small portion of m6A-positive sites (n = 310) were detected on all three platforms, potentially due to the read coverage threshold of 20 reads set by our tool, as well as variations stemming from techniques and experiments. Despite varying m6A landscapes at the site level, the distribution of m6A sites across genomic regions and motif frequency showed comparable results between all three methods (Figs. 3D and 3E), suggesting a high degree of reliability in m6ATM results.

### Stoichiometric estimation

**Figure 4:**
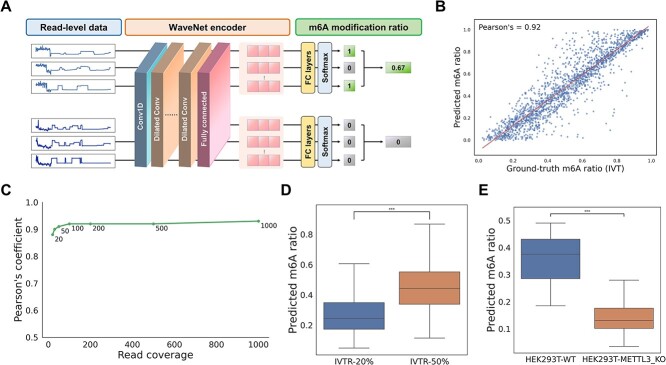
Stoichiometric estimation with m6A modification ratio. (A) Schematic diagram representing the proposed model for stoichiometric estimation, where the WaveNet-encoded read representations are fed into fully connected layers for read-level m6A prediction. The m6A modification ratio is calculated by averaging binary read-level labels of each site. (B) Correlation plot showing predicted m6A modification ratios from m6ATM and ground-truth values in *in silico* mixed IVT sites containing 20–1000 reads randomly. The result indicated a strong positive correlation between the two variables, with a Pearson correlation coefficient of 0.92. (C) Pearson correlation coefficients based on the number of reads used for prediction. (D) Predicted m6A modification ratios of high-confidence modified/unmodified sites (probability $\ge $0.9 or <0.5) in the IVTR datasets. Median ratios were 0.24 and 0.44 for the 20% and 50% modified groups, respectively. (E) Predicted m6A modification ratios of high-confidence modified/unmodified sites (probability $\ge $0.9 in WT, probability <0.1 in KO, and coverage $\ge $100) in the HEK293T datasets. Potential outliers with m6A ratios higher than 0.5 were removed from the analysis.

Stoichiometry of m6A can be a powerful indicator for understanding m6A biology, including identifying hotspot regions and dynamic regulation over time. Here, we established a simple neural network containing fully connected layers and a softmax layer for estimating m6A modification ratios. Specifically, WaveNet-encoded read representations were used as input for the neural network, which is a read-level classifier of m6A modification, to generate binary read-level labels (Fig. 4A and Figure S5). The m6A modification ratio was determined by averaging read-level labels, where each read was assigned a value of either 0 (unmodified) or 1 (modified). We trained the classifier with *in silico* mixed IVT sites, incorporating random percentages (0% to 100%) of m6A-modified and unmodified reads. The predicted m6A ratios showed a strong correlation (Pearson’s correlation coefficient = 0.92) with the ground-truth ratios (Fig. 4B). Additionally, we observed that read coverage had no substantial impact on model performance (Fig. 4C). Specifically, the correlation coefficients increased slightly from 0.88 to 0.93 as read coverage increased from 20 to 1000 reads.

To assess generalizability, we used the pre-trained classifier to analyze m6A stoichiometry in the IVTR dataset (Figure S1), synthesized with 20% or 50% m6A-modified nucleotides (Fig. 4D). High-confidence sites with predicted m6A probabilities above 0.9 or below 0.5 showed significant differences in m6A ratios between the 20% and 50% modified samples. The median predicted m6A ratios closely aligned with the ground-truth values, with medians of 0.24 and 0.44, respectively. Additionally, we applied our model to predict m6A stoichiometry in the HEK293T dataset, which exhibits varying levels of m6A modification depending on the presence or absence of METTL3 knockout. Consistent with IVT data findings, stoichiometric estimation in the HEK293T dataset confirmed a notable distinction between wild-type and METTL3 knockout samples, supporting the effectiveness of our model in predicting m6A stoichiometry (Fig. 4E).

### Case study: the m6A epitranscriptome in HepG2 cells

**Figure 5:**
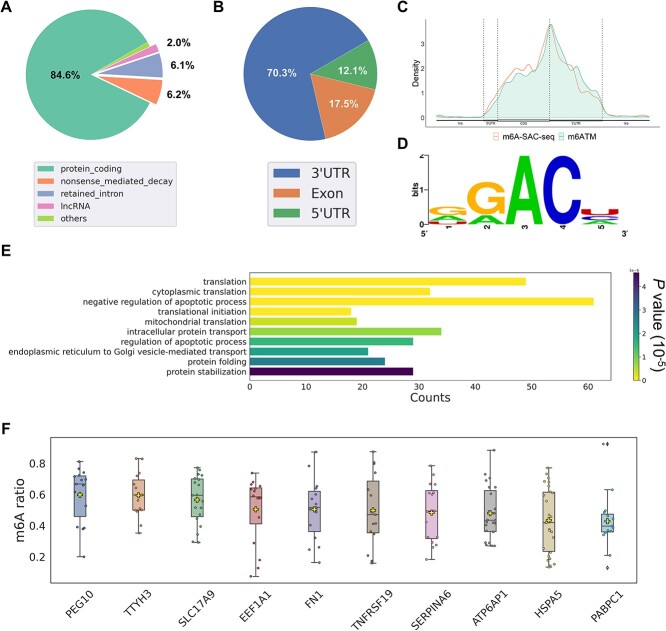
The m6A profile of HepG2 cells uncovered by m6ATM. (A) Distribution of 9631 HepG2 m6A sites at the transcript-level across transcript types. (B) Distribution of 8428 HepG2 m6A sites across genomic regions (5’ UTR, exon, and 3’ UTR). (C) Metagene plot showing the distribution of HepG2 m6A positive sites across genomic locations. (D) Motif distribution of HepG2 m6A sites. (E) GO enrichment analysis of 946 genes with high-confidence m6A sites in HepG2 cells. In the bar chart, the y-axis indicates the top 10 enriched GO terms ranked by *P* values, and “Counts” on the x-axis refers to the number of genes annotated to a specific GO term. The color of the bars represents the corresponding *P* value. (F) Top 10 gene transcripts ranked based on average m6A modification ratios in HepG2 cells. Only gene transcripts containing ¿15 m6A sites and 50 reads were included. The yellow cross symbol represents the mean m6A modification ratio.

Aberrant m6A modifications are implicated in cancer progression. To further investigate m6A dysregulation in liver cancer cells, we performed DRS on HepG2 cells and applied m6ATM to reveal a transcriptome-wide m6A landscape. From 1.89 million reads, m6ATM identified 8428 m6A positive sites from a total of 216 127 DRACH sites with at least 20 reads at gene-level (for transcript-level, 9631 m6A sites out of 297 029 DRACH sites). The majority of m6A sites were found in protein-coding transcripts (Fig. 5A), although a small population, about 2%, was observed in long noncoding RNAs. As expected, the predicted m6A sites were primarily concentrated in the 3’ untranslated region (3’ UTR), with significant enrichment near the stop codon (Figs 5B and 5C). Among the DRACH motifs, GGACU was identified with the highest frequency, representing $\sim $21.6% of the m6A sites (Fig. 5D and Figure S14). To investigate biological functions involving m6A regulation in HepG2 cells, we collected a list of 946 genes containing at least one m6A site and 100 reads for gene ontology (GO) enrichment analysis. (Fig. 5E). Several GO terms related to apoptotic regulation were significantly overrepresented, suggesting a potential role of m6A in cancer cell proliferation.

**Figure 6:**
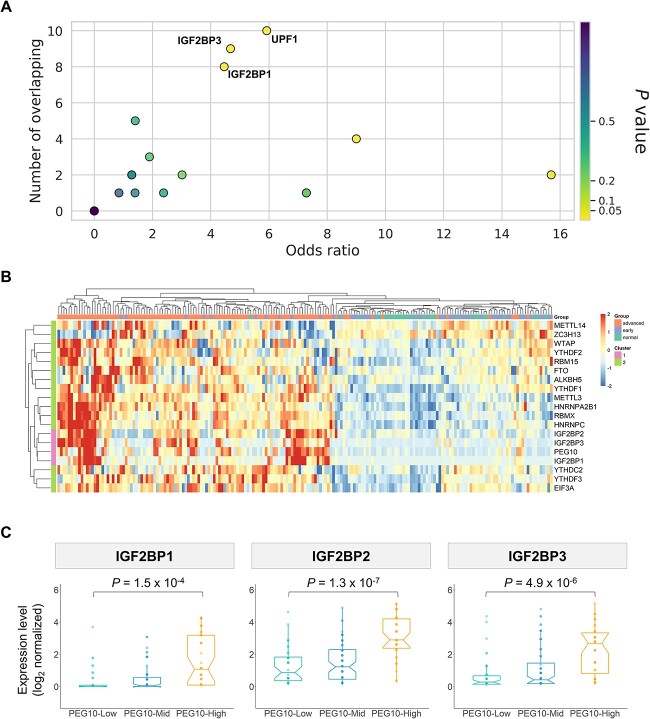
Enrichment of RBPs at predicted m6A sites on PEG10. (A) Significantly enriched RBPs near the PEG10 m6A peaks. *P* values were calculated using Fisher exact test. The number of overlaps between eCLIP-seq peaks and m6A peaks, and the odds ratio against unmethylated DRACH sites are shown in the scatter plot. (B) Heatmap illustrating expression profiles of m6A modulator genes across 191 HCC samples, including tumor and adjacent normal tissues. Row-scaled and log2-transformed FPKM values were used for analysis. The pink block on the left side of the heatmap indicates the cluster of IGF2BPs and PEG10, according to hierarchical clustering results. (C) Gene expression levels of IGF2BP1, IGF2BP2, and IGF2BP3 in 160 HCC tumor samples. Patients with HCC were categorized into three groups based on their PEG10 expression levels. The PEG10-High expression group comprised the top quartile of patients, the PEG10-Low expression group comprised the bottom quartile, and the remaining patients were assigned to the PEG10-Mid expression group.

To better understand candidate m6A-active genes associated with cancer development, we identified genes with ¿15 m6A sites and a top-tier average modification ratio (Fig. 5F). Among these highly m6A-modified genes, we focused on PEG10, a gene rarely found in normal liver tissues but significantly enriched in liver cancers, as demonstrated in previous research [42]. Notably, emerging evidence suggests that PEG10 upregulation is associated with cancer progression [42, 43]. To further explore the regulatory role of m6A on PEG10 transcripts, we analyzed the eCLIP-seq datasets of HepG2 cells from the ENCODE project, which provides information on RBP-binding sites of 103 RBPs. Interestingly, we observed significant enrichment of two m6A reader proteins, IGF2BP1 and IGF2BP3, near the m6A sites on PEG10 (Fig. 6A, Figure S7, and Figure S9). The IGF2BP1 binding region overlaps with 8 m6A sites, while the IGF2BP3 binding region overlaps with 9 m6A sites on PEG10 mRNAs. Given that IGF2BPs have been reported to enhance mRNA stability, we investigated the gene expression levels of IGF2BPs and PEG10 in patients with HCC. Short-read RNA-seq data from our previous study and the TCGA database consistently showed a positive correlation between PEG10 and IGF2BPs expression levels (Figs 6B, 6C, and Figure S10). Specifically, hierarchical clustering in the heatmap analysis revealed that IGF2BPs exhibited a highly similar gene expression pattern to PEG10 compared with other m6A modulators (Fig. 6B and Figure S10A). Additionally, samples with high PEG10 expression levels exhibited significantly elevated IGF2BPs expression levels (Fig. 6C and Figure S10B). These findings support the potential role of the m6A-IGF2BPs regulatory axis in modulating PEG10 transcript levels in HepG2 cells. Taken together, this case study demonstrates that m6ATM is effective in identifying potential m6A regulatory axes associated with cancer progression.

## Discussion

Building on the biological significance of m6A modifications in RNAs, we introduced a novel site-level m6A detection tool called m6ATM, which leverages informative DRS read data. In contrast to existing DRS-based methods, our tool relies on the long-term dependencies inherent in raw current intensity data for m6A detection. By integrating a read-level encoder WaveNet and a site-level aggregator DSMIL, m6ATM discerns subtle variations in modification patterns that may be ambiguous for other methods, as supported by benchmark tests with IVT and human cell line datasets.

We demonstrated that m6ATM achieved an ROC-AUC of 0.923, outperforming other tools in identifying m6A sites at a low modification ratio of 20% in IVT data. Although m6A is one of the most prevalent RNA modifications on mRNAs, prior research indicates that the modification ratio at individual sites is generally low, with a mean m6A modification level of $\sim $20% [5]. Therefore, the ability of detection tools to identify sites with low modification levels represents a crucial metric of practical significance. The low ratio of m6A modifications poses a challenge for site-level m6A prediction due to the potential masking effect of abundant unmodified reads on the relatively small fraction of modified reads. Consequently, an advanced aggregation approach for pooling read-level features is crucial when site-level m6A prediction is conceptualized as an MIL problem. In our study, we drew inspiration from the DSMIL model proposed in a previous study [30] and adapted it to effectively manage one-dimensional DRS data. The principal innovation of DSMIL is its dual-stream design with a trainable distance measurement, which enhances its ability to compute attention scores for handling unbalanced data structures. This strategy differs from the noisy-OR pooling layer employed in m6Anet, which uses a probabilistic framework to model read-level features. Notably, our results indicate that m6ATM outperformed m6Anet in predicting m6A modifications, as evidenced by a benchmark test conducted on a 20% m6A-modified IVT dataset. This finding reinforces the importance of applying DSMIL to site-level m6A prediction, even under conditions characterized by low m6A abundance.

It is conceivable that input data and feature encoding strategies can have a remarkable influence on model performance. In our model, we used not only signal data but also trace data for m6A prediction. Trace data produced by the recurrent neural network in the Nanopore Guppy basecaller model represent the base probabilities derived from ionic current data. Notably, it has been used by another neural network in the basecaller to infer nucleotide sequences within the nanopore channel. We suggest that trace data provide refined information for differentiating the modification patterns of ionic currents. To the best of our knowledge, no existing m6A detection tool uses trace data in a prediction model. One previous study [44] evaluated the performance of a pseudouridine prediction model using different combinations of DRS read features, including current intensity, dwell time, and trace. Their results indicated that the combination of current intensity and trace data was more effective than other combinations in predicting pseudouridine sites, consistent with our m6A analysis findings (Fig. 2C and Figure S4).

To optimize the model, it is necessary to match the input data with an appropriate neural network. Given the similarities between nanopore current intensity data and audio data, we propose using WaveNet, a model tailored for audio data processing, as an effective solution for handling sequential signals and trace data. Since its introduction in 2016, WaveNet has shown great promise in a wide range of audio-related tasks, such as musical note generation and heart-sound classification [45, 46]. The architecture of the dilated convolutional layers in WaveNet enables it to have a large receptive field for effectively capturing complete waveforms and disruptions in sequential data while maintaining a relatively low number of model parameters. This design is particularly advantageous for modeling the long-term dependencies of the read-level features in our model. According to the results of our model performance evaluation (Figs 2 and 3), we are confident that the application of WaveNet in our model allows for sophisticated feature extraction of signal and trace data.

As several previous studies have reported varying m6A landscapes between different detection strategies [20, 47], we also observed that the number of m6A sites in HEK293 cells detected by our tool, as well as by the other two methods, was limited. In addition to variations attributable to techniques and experiments, it is important to acknowledge that differences in mRNA expression levels across datasets also contribute to the observed variety of m6A landscapes, owing to the minimum read coverage of 20 reads required in our model. Specifically, we found that 47% of the identified m6A sites in miCLIP-seq and 75% in m6A-SAC-seq could not be profiled for m6A information using our model because of the low abundance of covering reads (Figure S12). This finding implies that increasing sequencing throughput may be necessary to provide more comprehensive detection results and reduce the variation between different methods.

A growing number of studies have reported a significant role of m6A modifications in various cancers [48, 49]. For example, overexpression of the m6A methyltransferase METTL3 promotes cancer cell proliferation and migration in HCC, leading to poor prognosis in patients with HCC [50]. However, the underlying mechanism remains unclear. In this context, we applied our m6A prediction model to investigate active m6A-modified regions in HepG2 cells. Our findings revealed a total of 8428 m6A sites across 3304 genes. While most genes exhibited one or two m6A modifications (Figure S13), PEG10 stood out with a high number of m6A sites (n = 15) and elevated modification ratios, suggesting it as a candidate for aberrantly m6A-modified transcript associated with cancer progression. Consistent with earlier studies [42, 43], we found that PEG10 levels were significantly upregulated in overt HCC samples (Figure S8). This abnormal upregulation was supported by the observed enrichment of IGF2BPs, which are known to enhance mRNA stability, in the m6A-modified regions of PEG10. Intriguingly, a recent study highlighted the important role of IGF2BP1 in m6A-mediated PEG10 upregulation in endometrial cancer [43], reinforcing evidence for the m6A-IGF2BPs-PEG10 regulatory axis identified in our study. Despite the limitation of low resolution arising from MeRIP-seq in their study, the authors reported that a specific m6A site (Chr7: 94668076) within the 3’UTR of PEG10 may play a pivotal role in this regulation. In accordance with this finding, the same m6A modification site was identified in our analysis, with a high modification ratio. Further investigation is necessary to understand the functional implications of this m6A site in liver cancer development. Collectively, we are confident that the preliminary findings of our m6A analysis provide a solid foundation for future research.

Although our model provides robust performance in m6A detection, several limitations need to be addressed for future advancements. First, m6A sites in non-DRACH motifs could not be detected. According to previous studies, $\sim $20% of m6A sites are located in non-DRACH motifs [6]. The inability to profile non-DRACH m6A sites suggests that valuable insights into the regulation of m6A may have been missed. However, incorporating m6A prediction of non-DRACH motifs into the model may introduce substantial noise. Consequently, a specialized denoising strategy is required to manage voluminous data effectively. Second, the secondary structure of RNA was not considered in our model. Prior research reported that m6A modifications alter the local structure of RNA to regulate protein accessibility [51, 52]. Therefore, information on RNA secondary structure can provide valuable clues for m6A prediction. Additionally, one study showed that combining chemical probes with Nanopore sequencing enabled the capture of structural information using ionic current data [53]. These findings merit future investigation to explore how structural information can be leveraged in m6A prediction models. Finally, a more extensive scale of training data may be needed to improve the model performance. Although our pretrained model using IVT training datasets performed well in other non-IVT datasets, such as human cell line data, using a broader variety of datasets could further enhance its generalizability. Unlike IVT data, it is challenging to obtain balanced training data from cell line datasets. The number of positive training examples may be limited and concentrated on certain motifs. Implementing an appropriate data augmentation strategy or using extremely high-throughput sequencing data could provide promising solutions to address this challenge.

## Conclusion

To bridge the gap in transcriptome-wide m6A prediction, we developed a novel DRS-based computational method called m6ATM. Our results demonstrated that by incorporating a WaveNet encoder and a DSMIL model, m6ATM was effective in extracting discriminative features from long-term dependencies in nanopore ionic currents at the read level, achieving high accuracy in site-specific m6A prediction. The effectiveness of m6ATM was validated across diverse datasets, including IVT and human cell line data, where it outperformed existing DRS-based methods, especially at sites with a low m6A modification frequency. A case study of m6A site detection in HepG2 cells further highlighted the potential of m6ATM as a robust tool for advancing m6A-related biological research. Approximately 8400 m6A sites were identified across a broad range of genes, including those involved in cancer cell apoptotic pathways. Additionally, we uncovered the potential role of the m6A-IGF2BPs-PEG10 regulatory axis in liver cancer cells and HCC samples, underscoring this method’s capability to provide new insights into cancer biology. However, further investigations are necessary to fully elucidate the underlying mechanisms. In summary, the method proposed in this study offers a reliable and user-friendly tool for obtaining m6A epitranscriptomes for biological research.

**Key Points**

- In this study, we present m6ATM, a Direct RNA Sequencing and deep neural network-based m6A detection tool without the need for additional third-party resquiggling tools.
- m6ATM demonstrated superior performance compared with other m6A detection tools across diverse validation datasets.
- Our findings in the HepG2 m6A epitranscriptome analysis showed m6ATM can be a reliable tool for conducting m6A-related biological research.
- We identified the potential role of the m6A-IGF2BPs-PEG10 regulatory axis in liver cancer cells; however, additional research is necessary to clarify the underlying mechanisms.

## Supplementary Material

supplementary_bbae529

## Data Availability

Raw Nanopore Direct RNA sequencing datasets generated in this study are available at GSE265754 (https://www.ncbi.nlm.nih.gov/geo/query/acc.cgi?acc=GSE265754) for IVT data and GSE265867 (https://www.ncbi.nlm.nih.gov/geo/query/acc.cgi?acc=GSE265867) for HepG2 data.

## Code availability

Source codes of m6ATM can be obtained at https://github.com/poigit/m6ATM. Source codes used for analyses in this paper can be obtained at https://github.com/poigit/m6ATM_analysis.
