# Supplementary material for: m6ATM: a deep learning framework for demystifying the m6A epitranscriptome with Nanopore long-read RNA-seq data
Source: Brief Bioinform. 2024 Oct 22;25(6):bbae529. doi: 10.1093/bib/bbae529 (PMC11495873; doi:10.1093/bib/bbae529)
Supplement: supplementary_bbae529 [file supplementary_bbae529.docx]

**Supplementary Methods**

**In-vitro transcription and barcoding**

We modified the protocol previously described by Liu et al. [1] to generate a different IVT dataset, designated as IVTR. In addition to the originally designed four IVT templates, we newly introduced ten reference templates featuring additional 559 DRACH motifs in the IVTR dataset. We then followed the in-vitro transcription steps in their publication but replaced the m6ATP materials with a mixture of ATP and m6ATP in the m6A-modifed templates to mitigate the occurrence of densely aligned m6A nucleotides in neighboring regions. To prepare the samples for DRS, all IVT products were subjected to 5' capping and polyadenylation. The final products contain sparsely modified sequences with either 20% or 50% m6A nucleotides, or all unmodified adenosines (**Figure S1 and Table S1**). For multiplex sequencing, IVTR-20% and IVTR-50% samples were additionally ligated to unique barcodes as detailed in [1] and then demultiplexed using DeePlexiCon described in the same publication.

**RNA extraction and purification**

Total RNAs were extracted from HepG2 cells using the standard TRIzol (Invitrogen) protocol. Briefly, cells were homogenized in 1 ml of TRIzol reagent and incubated at 4°C for 10 minutes. Next, the total RNA precipitate was collected as pellets after appropriate centrifugation. RNA pellets were then washed with 75% ethanol and resuspended in RNase-free water for further use. To purify poly-adenylated RNAs, the kit NEBNext® Poly(A) mRNA Magnetic Isolation Module (NEB #E7490S/L) was used. All steps were performed according to the manufacturer’s instructions to isolate intact poly(A)+ RNAs with Oligo dT Beads d(T)_25_. Last, concentrations and purity were measured by the NanoDrop ND1000 Spectrophotometer and the Qubit Fluorometer to ensure the quality of harvested RNAs.

**Library preparation and sequencing**

500 ng of poly(A)+ RNAs extracted from IVT and HepG2 samples were used for Direct RNA Sequencing on the MinION/PromethION sequencers (Oxford Nanopore Technologies Ltd., ONT). All RNA libraries were prepared using the RNA-SQK002 Kit (DRS_9080_v2_revQ) and sequenced on R9.4.1 flowcells, following the guidelines provided by ONT (**Table S1**).

**Ground-truth datasets**

For IVT data, we used two distinct validation datasets: in-silico mixed IVT data and IVTR data (**Figure S1**). In case of in-silico mixed IVT data, reads were randomly sampled from 100% m6A-modified and unmodified datasets to generate IVT sites with varying m6A modification ratios (0 - 100%). On the other hand, IVTR data consists of sparsely modified sequences using only 20% or 50% m6A during template synthesis. For human cell-line data, we used the publicly available m6A-SAC-seq data of HEK293 cells from NCBI GEO repository under accession number GSE162356 as the validation data [3]. For further comparison with traditional short-read data, miCLIP-seq data was retrieved from NCBI GEO repository under accession number GSE63753 [4].

**Implementation of other DRS based tools**

For the benchmark test, we executed the following published DRS-based m6A detection tools according to their instructions. Unless otherwise specified, we used the default parameters for all tools.

EpiNano (https://github.com/novoalab/EpiNano)

EpiNano version 1.2 offers two different prediction modes: EpiNano-Error and EpiNano-SVM. We ran the EpiNano-SVM mode for the benchmark test. To ensure optimal basecalling performance for EpiNano, we used the recommended Guppy version 3.1.5. The m6A prediction was performed by using the pretrained SVM model “rrach.q3.mis3.del3.linear.dump” provided by EpiNano.

m6Anet (https://github.com/GoekeLab/m6anet)

m6Anet is a deep-learning-based method. For transcriptome-wide m6A detection, we ran m6anet inference by the default pretrained model trained on human HCT116 cell lines.

Tombo (https://github.com/nanoporetech/tombo)

Tombo re-squiggles signal data from raw Nanopore reads and detects diverse modified bases in samples. For modification prediction, Tombo can detect aberrant signal patterns from expected canonical bases in the “de_novo” mode or identify the difference between two samples in different modification levels using the “level_sample_compare” mode. Here, we ran Tombo in the “de_novo” mode to obtain the fraction of modified reads for each site.

MINES (https://github.com/YeoLab/MINES)

MINES uses fraction modification values predicted by Tombo as input for the Random Forest m6A detection model. Since the original output of MINES only includes 4 instances of 18 DRACH motifs, we have adopted the same strategy as outlined in [2] to customize the Python script “cDNA_MINES.py” and generate prediction results for all DRACH motifs.

m6ABasecaller (https://github.com/novoalab/m6ABasecaller)

m6ABasecaller incorporates a m6A basecalling model that can predict m6A in addition to canonical four bases (A, C, G, T, and m6A) into Guppy basecaller. We performed m6A basecalling with the default model “rna_r9.4.1_70bps_m6A_hac.cfg” and used the recommended [ModPhred](https://modphred.readthedocs.io/en/latest/) pipeline to obtain site-level reports by adding “ --minModFreq 0”.

**Supplementary Figures and Tables**


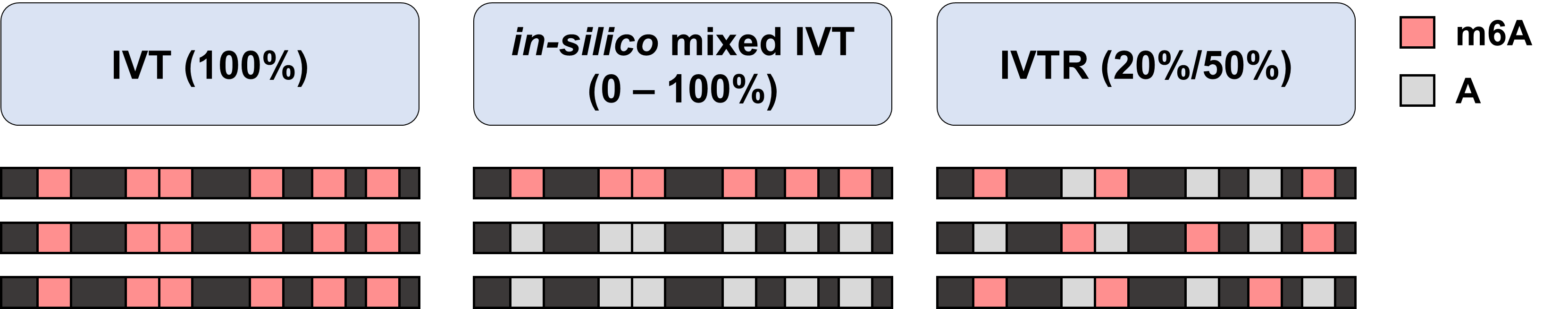


**Figure S1 Design of site-level IVT data.**

Three different IVT datasets were used in this study: IVT (100%), in-silico mixed IVT (0 - 100 %), and IVTR (20%/50%). IVT (100%) data consists of only m6A-modifed adenosines, while in-silico mixed IVT data is a mixture of reads containing only m6A-modifed adenosines or only unmodified adenosines. Last, IVTR data contains 20% or 50% m6A-modifed adenosines in templates. In addition, these modified adenosines in IVTR sequences are randomly distributed across reads.

**
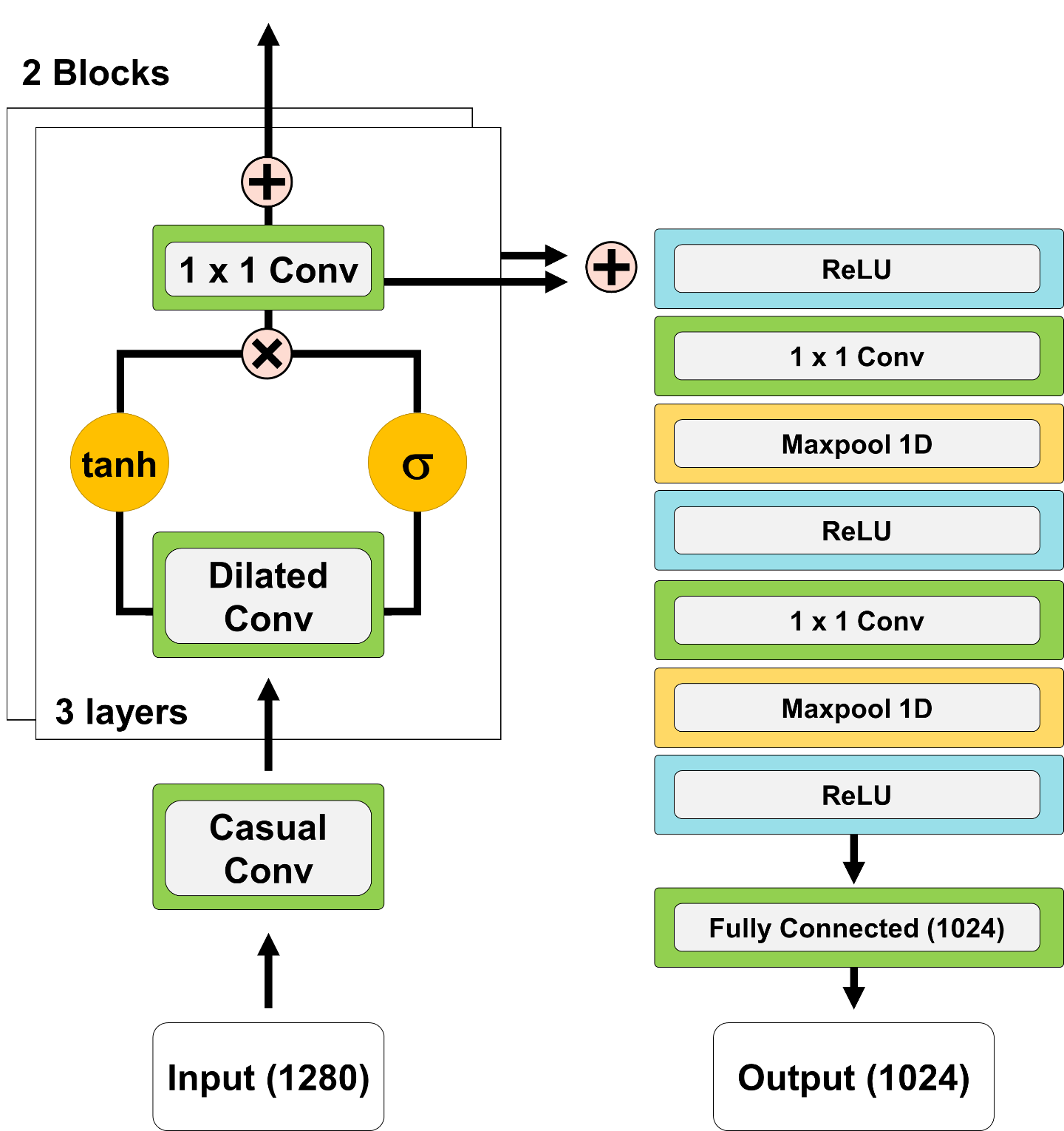
**

**Figure S2 Modified WaveNet architecture for feature encoding.**

Input data of length 1280 is fed into a lightweight WaveNet model consisting of 2 blocks and 3 layers and passed through fully connected layers to generate output data of length 1024.


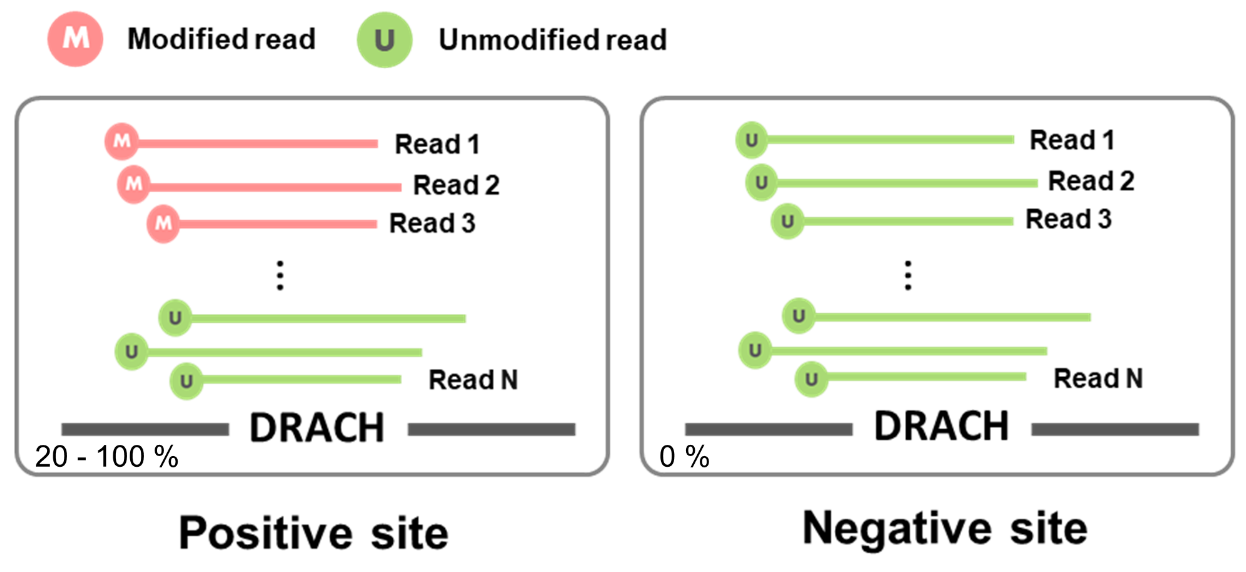


**Figure S3 Training data used in the m6A prediction model.**

Training datasets include in-silico mixed IVT data with 20 - 100% m6A modified reads (positive site) and 0% modified reads (negative site).


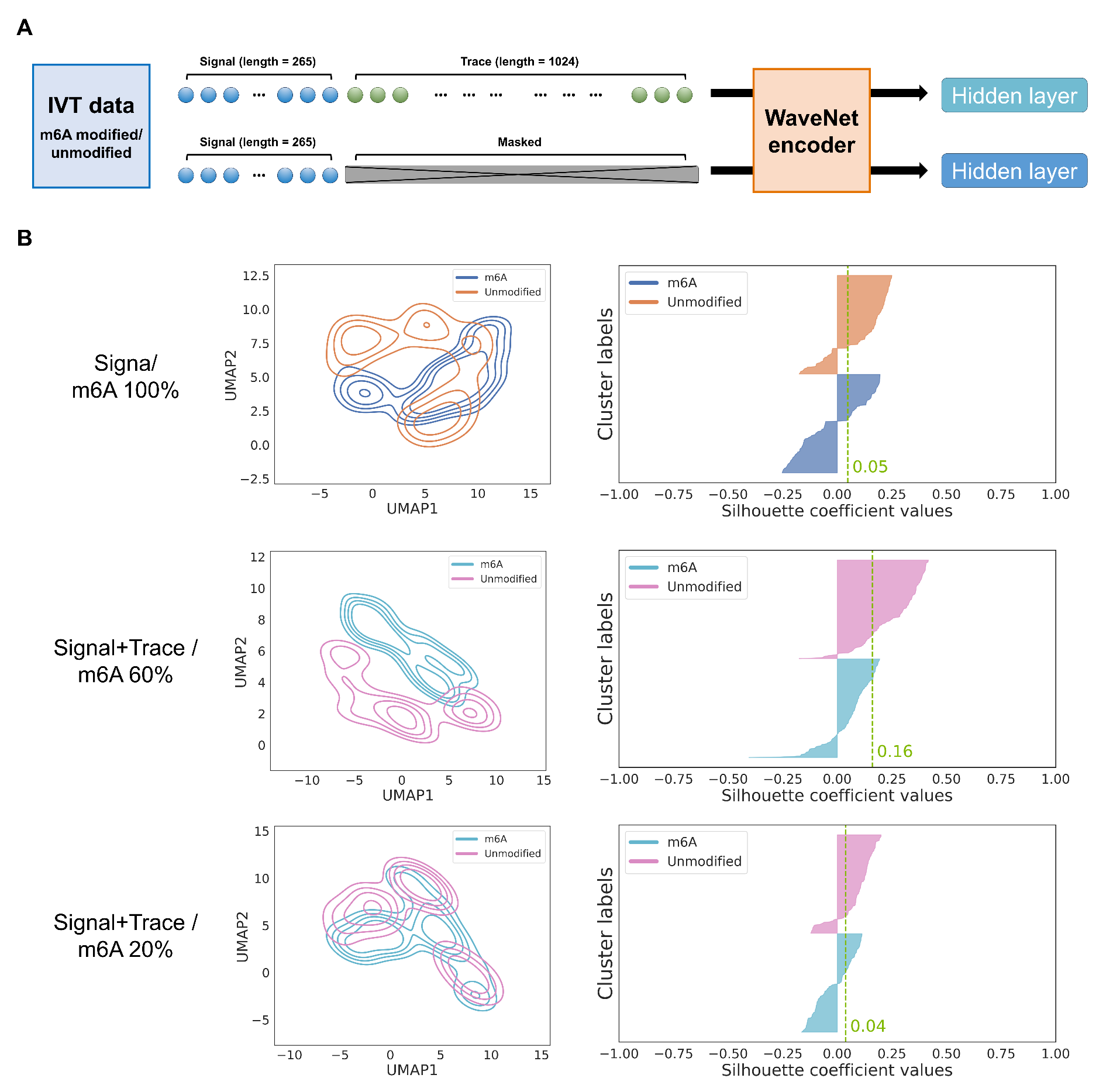


**Figure S4 Site-level feature effectiveness at 191 IVT sites in different conditions.**

(A) Schematic diagram of two feature extraction strategies: one uses a combination of signal and trace data as input, while the other one uses only signal data. (B) The contour plot of UMAP-transformed site-level features and at 191 DRACH sites (left) and the Silhouette plot of UMAP clusters (right) in different m6A modification conditions or different input data.


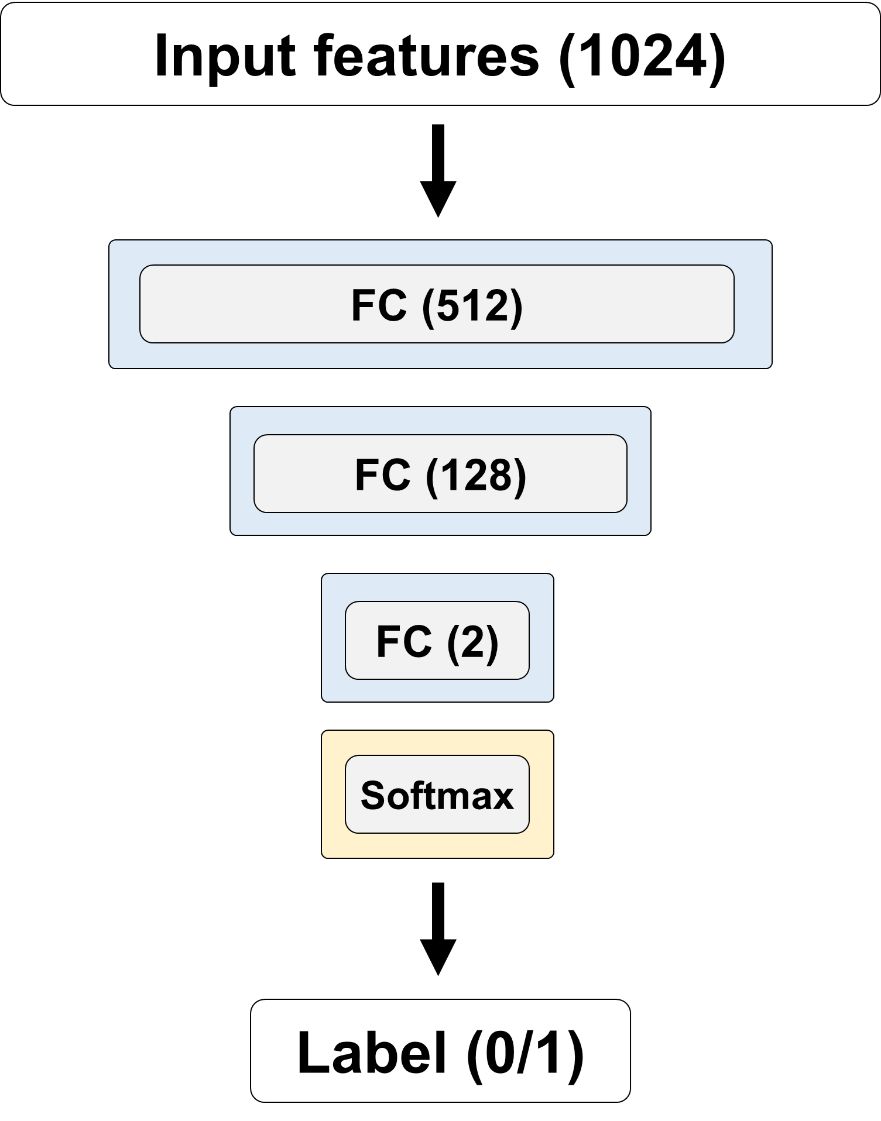


**Figure S5 Read classifier for stoichiometric estimation.**

The read classifier consists of three fully connected layers and a Softmax layer. It is applied to transform input WaveNet encoded features of length 1024 to read-level pseudo-labels of 0 or 1.


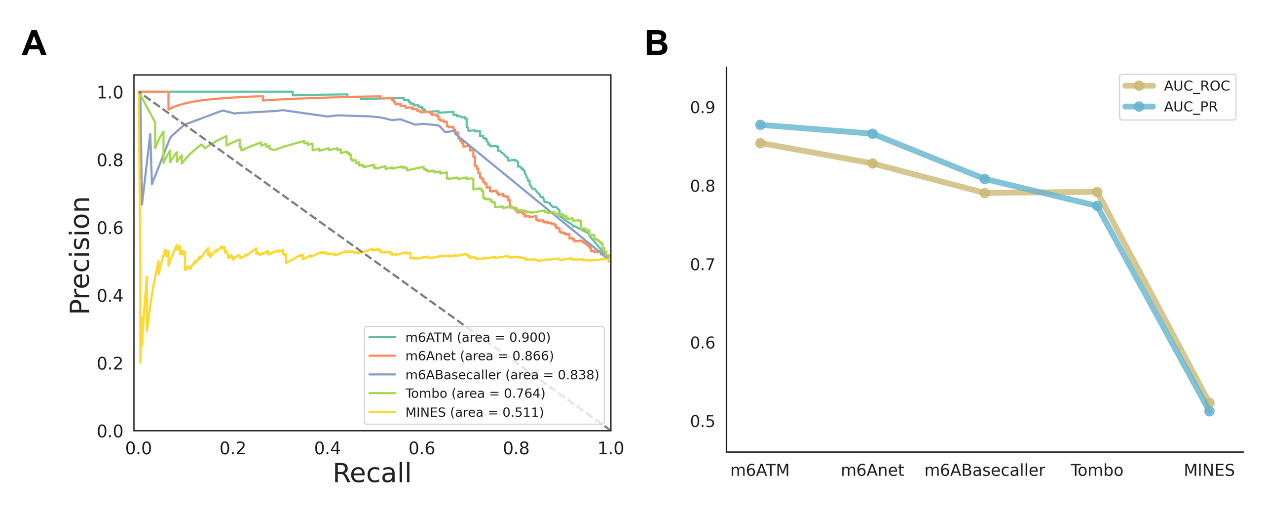


**Figure S6 Benchmarking with other DRS-based methods using HEK293 data**

(A) Precision-recall curves for m6A prediction of HEK293 cells by using DRS based tools. Only sites that meet the following two conditions were included: (1) at least 50 read coverage (2) detectable by all methods. All 570 sites were used for validation, including 285 positive sites identified from previously reported m6A sites in the m6A-SAC-seq data and 285 negative sites randomly sampled from sites not reported as m6A-modified. EpiNano was excluded from the analysis because it could not complete the job within two weeks. (B) Mean AUC scores for the ROC and PR curves from 10 repeated analyses using different negative sites.


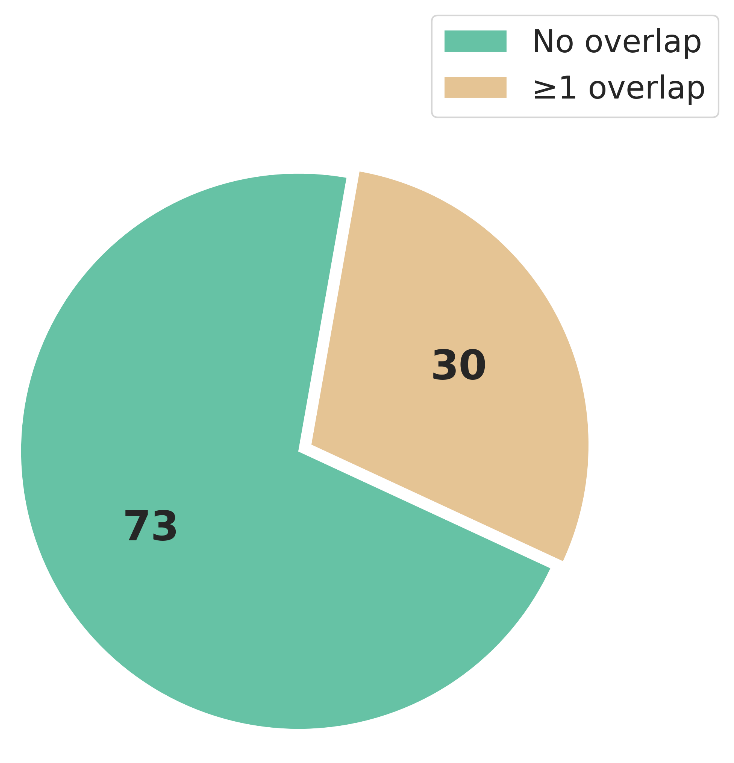


**Figure S7 RNA binding proteins bound to DRACH sites on PEG10 mRNA.**

The pie chart shows the presence or absence of eCLIP-seq peaks within the DRACH sites on the PEG10 gene transcript. Among 103 RNA binding proteins, 30 of them bind to at least one DRACH site.


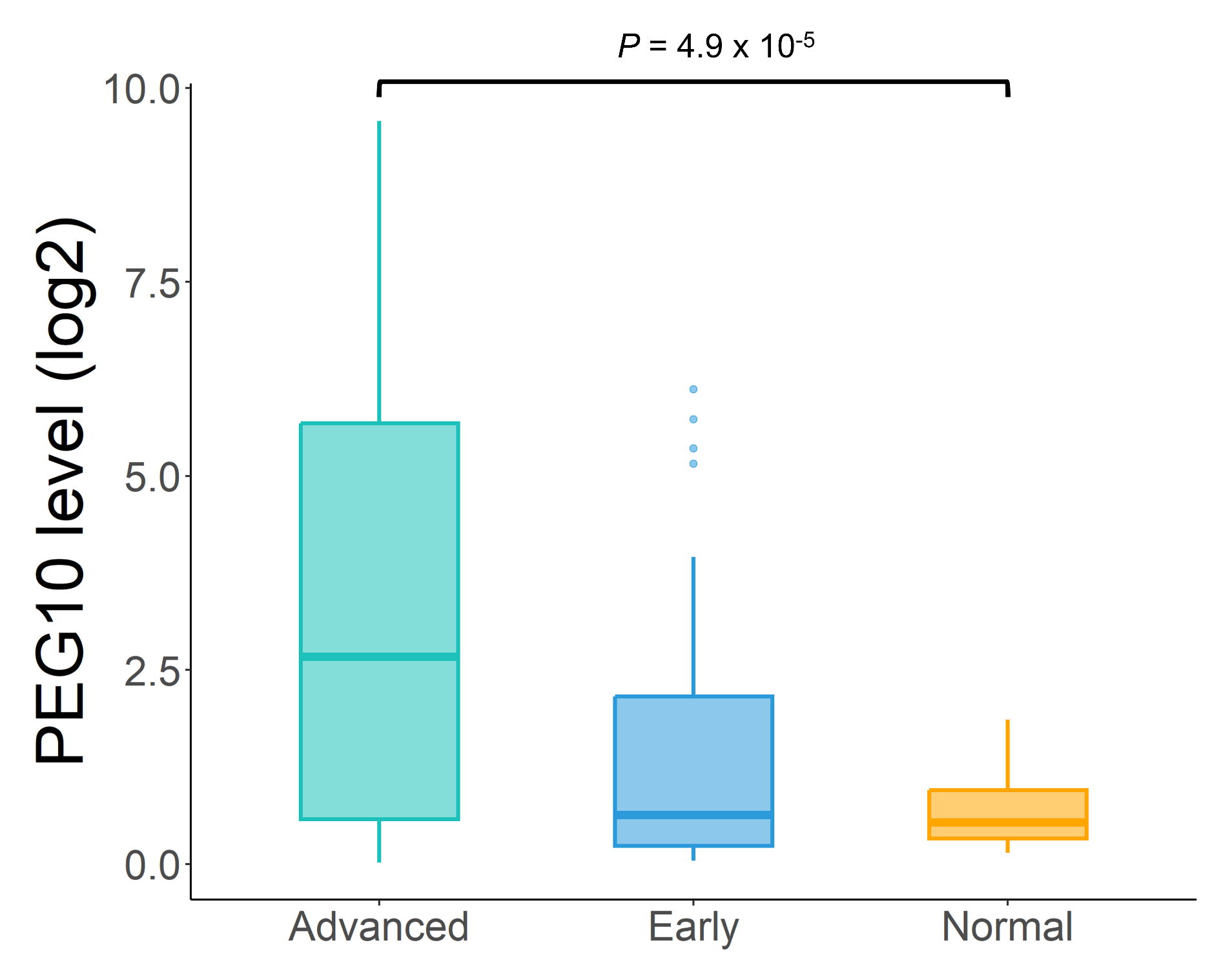


**Figure S8 PEG10 gene expression level in HCC at different stages.**

PEG10 gene expression levels (log_2_ FPKM) from 191 HCC samples, including 108 overt, 52 early, and 31 adjacent normal samples. The Wilcoxon signed-rank test was used to evaluate the difference between overt HCC samples and adjacent normal samples.


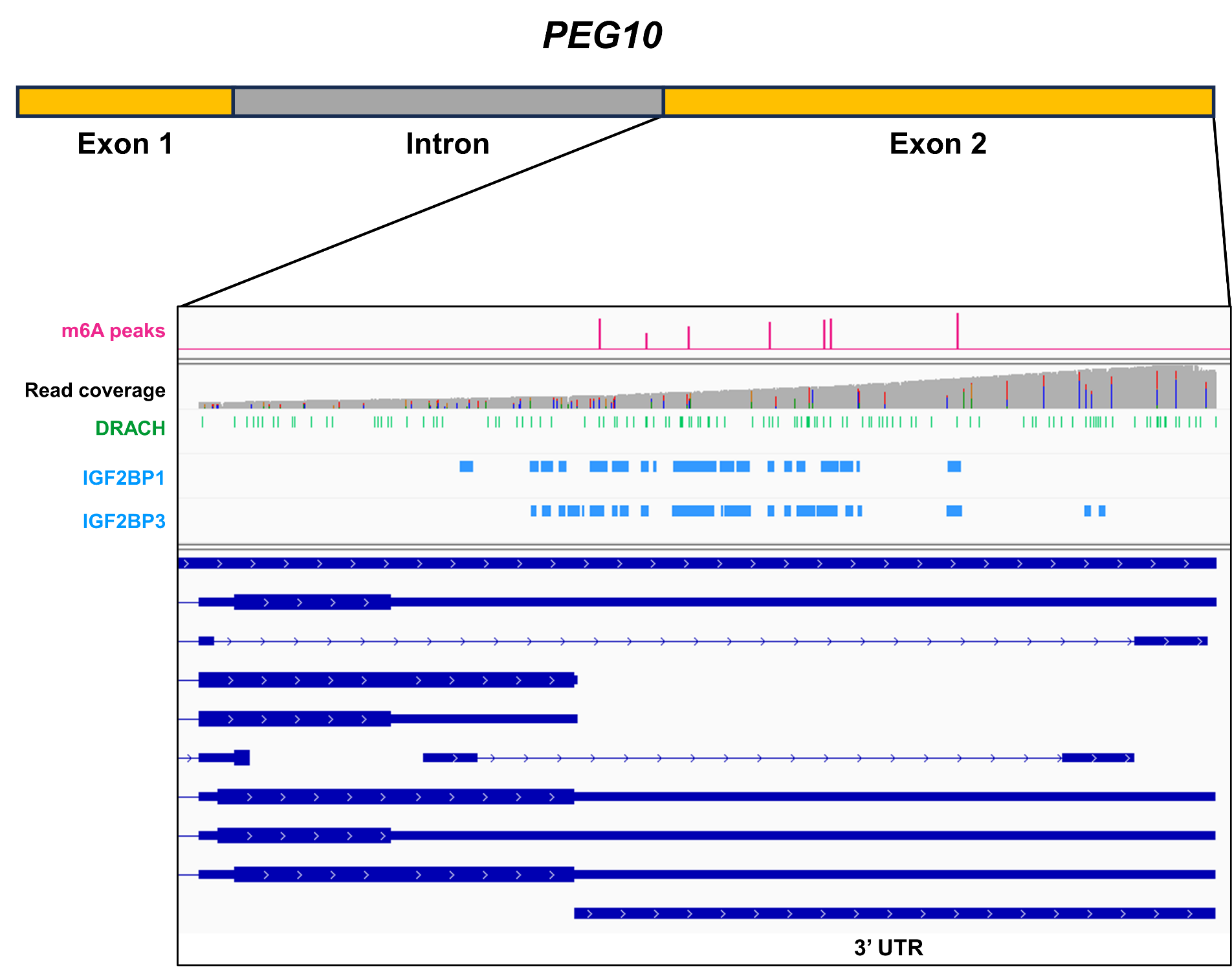


**Figure S9 The m6A sites overlapped with RNA binding protein binding sites on PEG10.**

Seven m6A peaks overlapped with eCLIP-seq peaks of IGF2BP1 and IGF2BP3 on PEG10 exon 2 are displayed by the IGV snapshot. Pink bars represent the m6A modification ratio, ranging from 0 to 1, at each site. Green bars represent the position of DRACH sites. Blue blocks show the RNA binding protein binding regions.


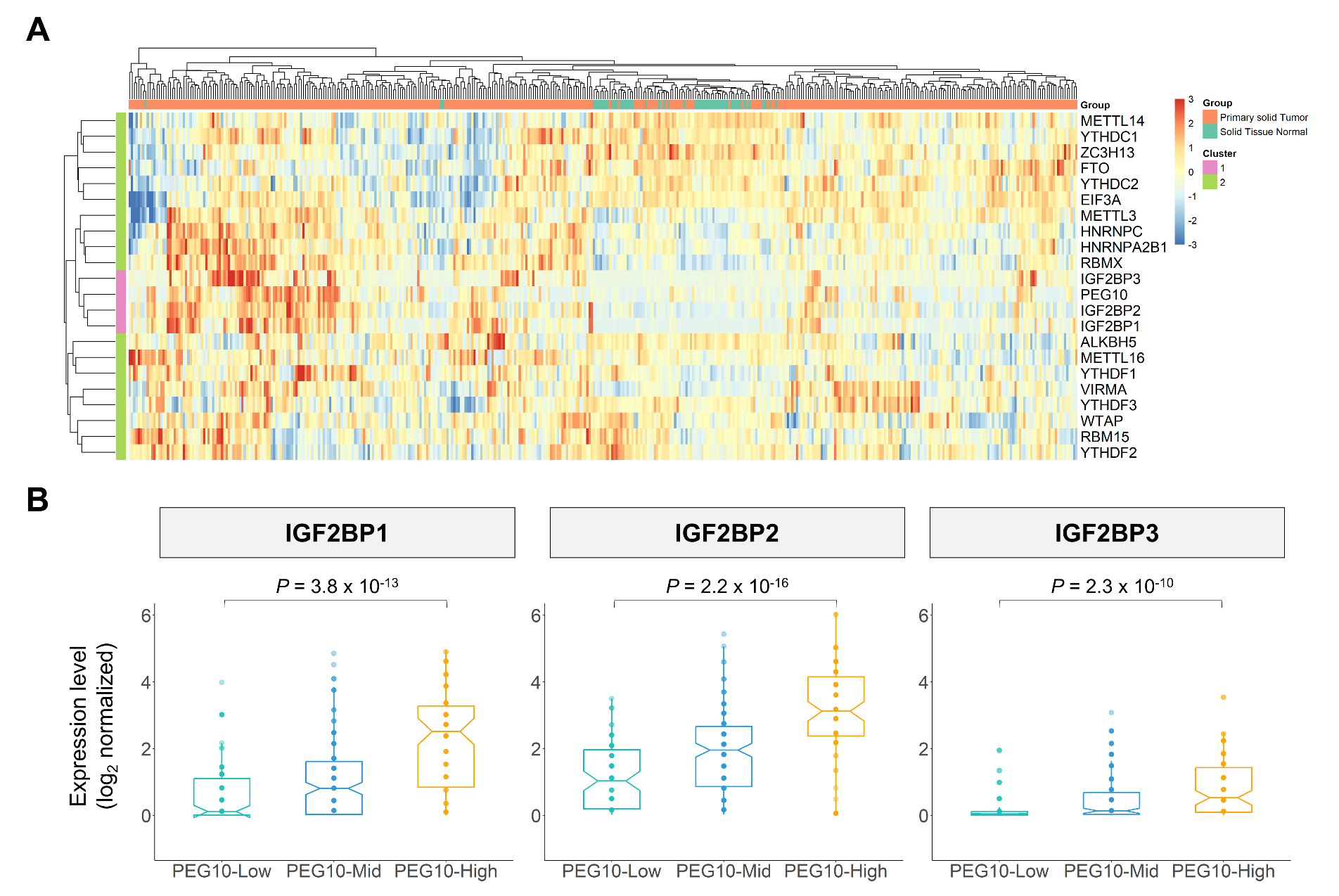


**Figure S10 Expression analysis of PEG10 and IGF2BPs in TCGA-LIHC datasets.**

(A) Heatmap illustrating expression profiles of m6A modulator genes in samples from 421 HCC patients, including tumor and adjacent normal tissues. Row-scaled and log2-transformed read counts were used for analysis. The pink block on the left side of heatmap indicates the cluster of IGF2BPs and PEG10 according to hierarchical clustering results. (B) Gene expression levels (log_2_ FPKM) of IGF2BP1, IGF2BP2, and IGF2BP3 in tumor samples derived from 371 HCC patients. HCC patients were categorized into three groups based on PEG10 expression levels. The PEG10-High expression group consisted of the top quarter of patients, while the PEG10-Low expression group consisted of the bottom quarter. The remaining patients were categorized into the PEG10-Mid expression group.


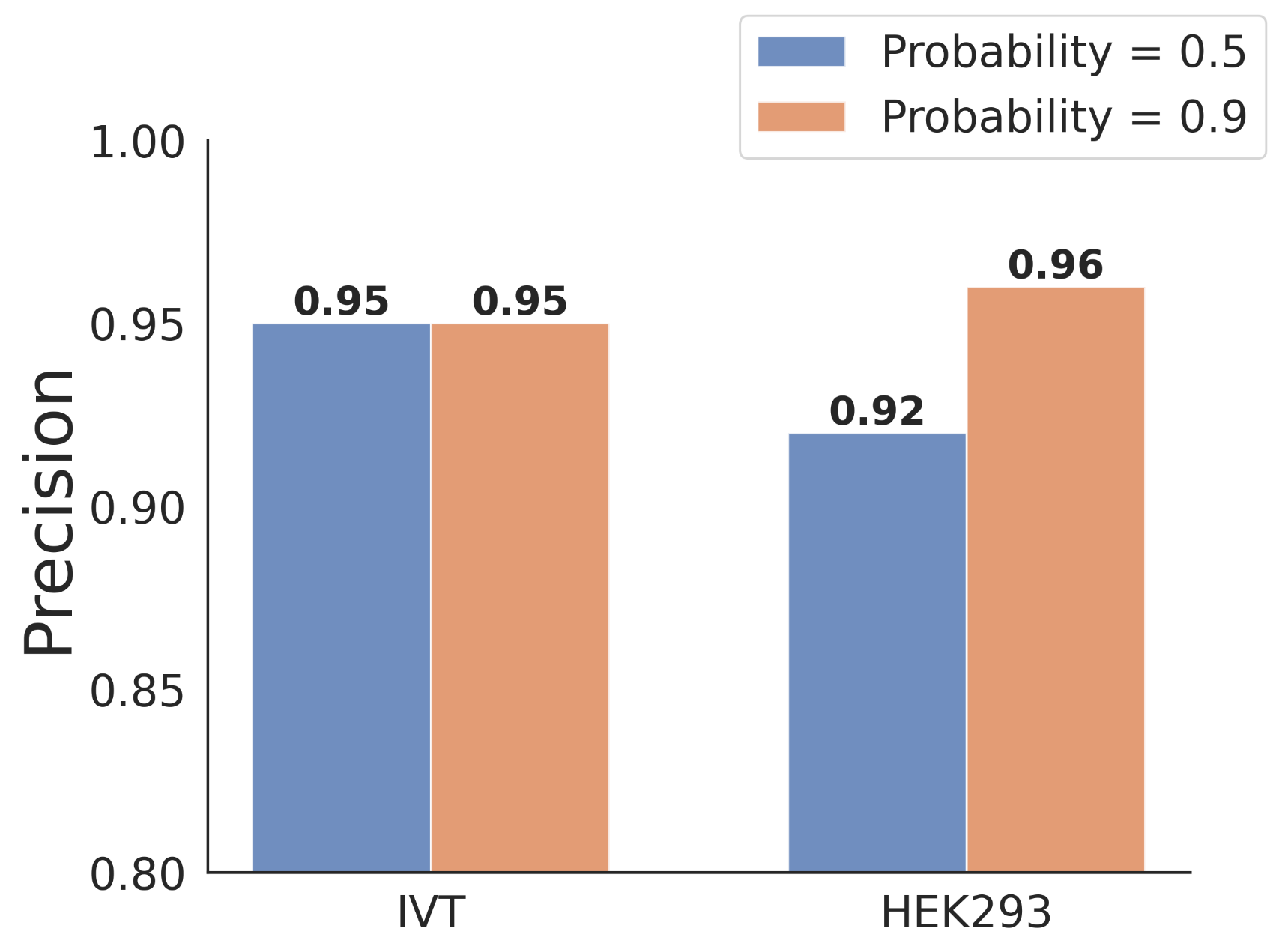


**Figure S11 Precision values under different probability thresholds.**

Different probability thresholds (*P* = 0.5 or 0.9) were applied for m6A detection in the IVT and HEK293 datasets, with a precision value reported for each group.


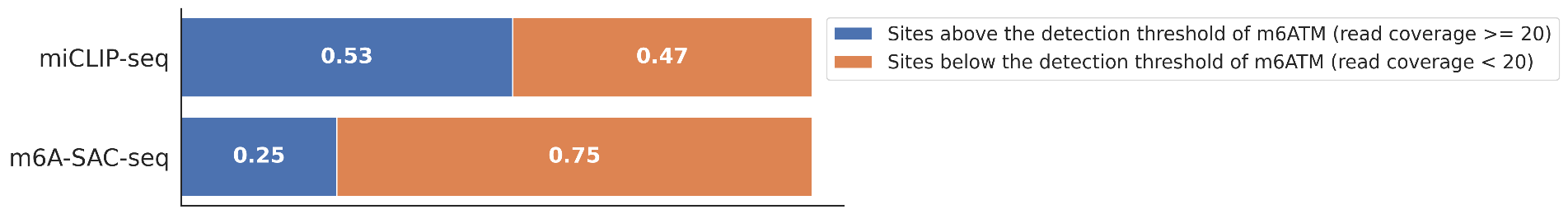


**Figure S12 The m6A sites below the detection threshold of m6ATM**

The proportion of m6A sites (identified by other methods) that exhibit less than 20 read coverage in DRS data. Therefore, these sites are not available for m6A profiling by using our tool.

**
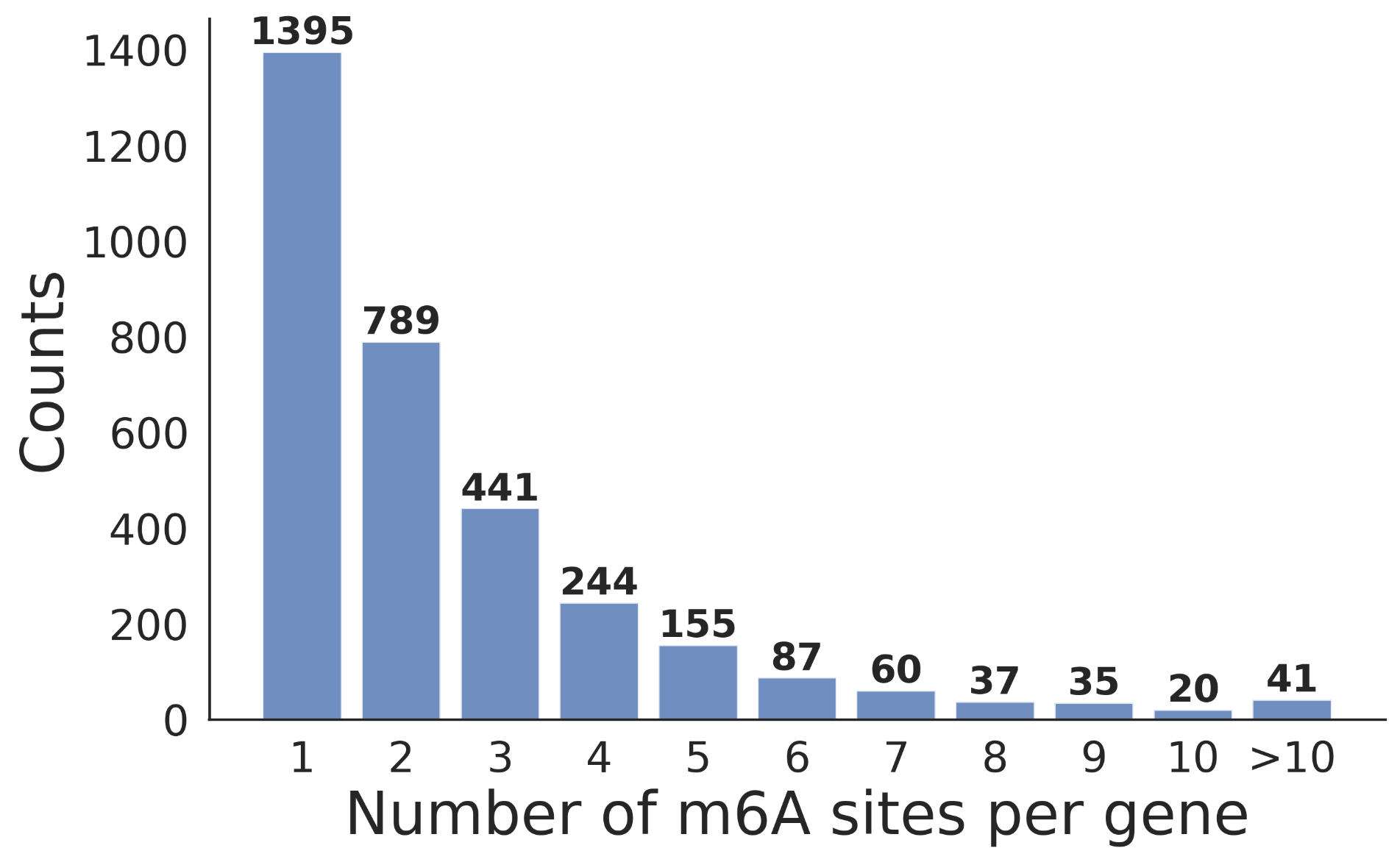
**

**Figure S13 Frequency distribution of genes based on the number of m6A sites in HepG2 cells.**

The bar chart shows the frequency of genes based on the number of m6A sites present in each gene.

**
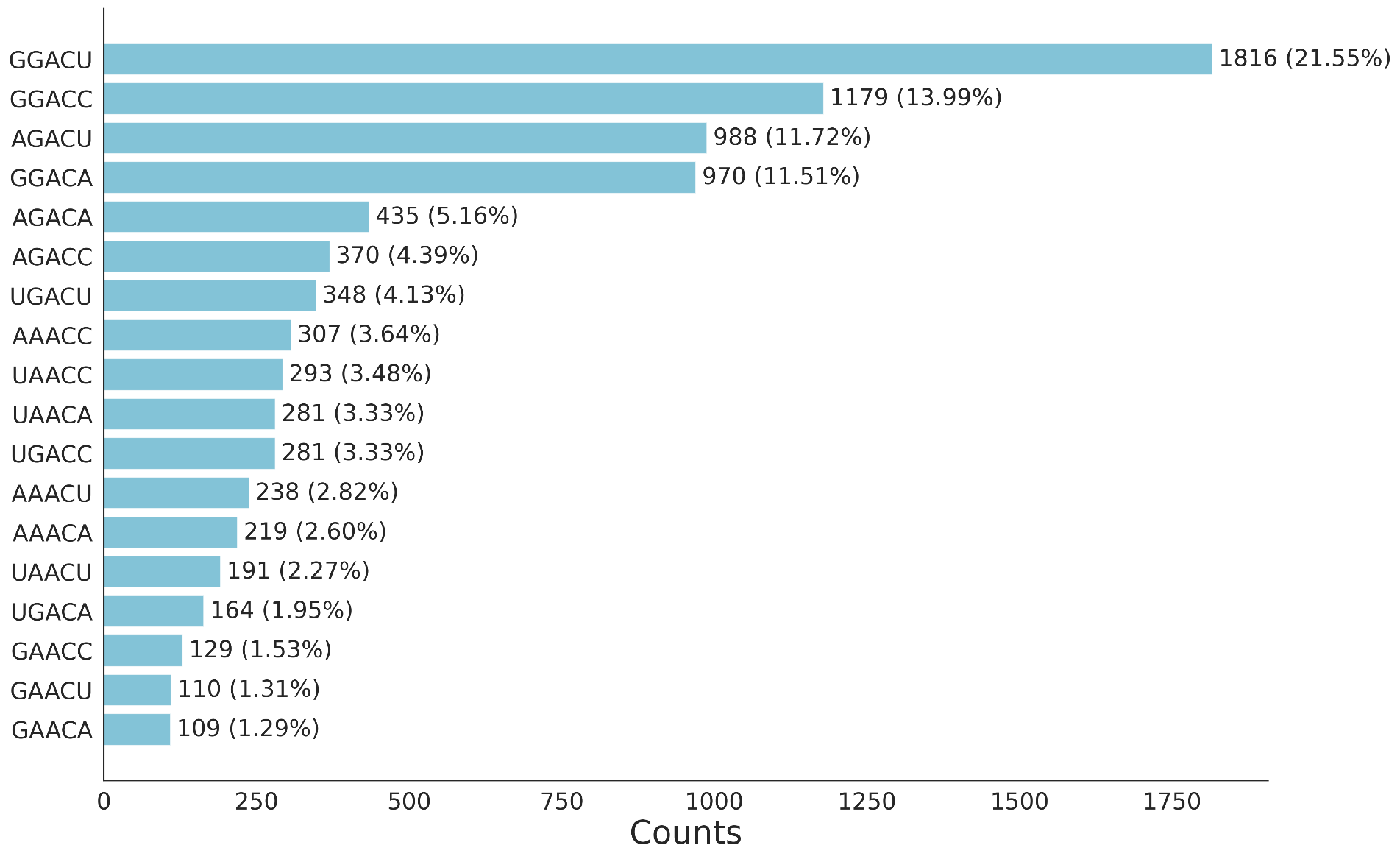
**

**Figure S14 Frequency distribution of m6A sites in HepG2 cells across**

**different DRACH motifs.**

The bar chart shows the frequency of m6A sites across 18 different DRACH motifs.

**Table S1 Direct RNA Sequencing datasets**

|  | Protocol | Sequencer | Num. of reads |
| --- | --- | --- | --- |
| IVTR-unmodified | SQKRNA-002 | MinION | 294,594 |
| IVTR-20% m6A | SQKRNA-002 | MinION | 147,100 |
| IVTR-50% m6A | SQKRNA-002 | MinION | 108,967 |
| HepG2 | SQKRNA-002 | PromethION | 1,889,614 |

**Table S2 Computational resource assessment**

We executed m6ATM on a 96-core local server setup with CentOS 8 operating system to test computational resource usages of a mini testing dataset containing 50,000 IVT reads and a cell-line dataset containing approximately 1 million reads. For multiple processing, we configured the number of processes to be 24. Additionally, we ran it on the GPU environment powered by the NVIDIA A100 Tensor Core GPU 40GB.

| *m6atm run* | Num. of reads | Runtime | Peak memory/CPU |
| --- | --- | --- | --- |
| IVT | 50,000 | ~ 19 min | 8.7 Gb |
| HEK293 | 1,179,256 | ~ 16.5 hr | ~ 30 Gb |

**References**

1. Liu H, Begik O, Lucas MC, Ramirez JM, Mason CE, Wiener D, Schwartz S, Mattick JS, Smith MA, Novoa EM: **Accurate detection of m6A RNA modifications in native RNA sequences**. *Nature communications* 2019, 10(1):1-9.
2. Zhong Z-D, Xie Y-Y, Chen H-X, Lan Y-L, Liu X-H, Ji J-Y, Wu F, Jin L, Chen J, Mak DW: **Systematic comparison of tools used for m6A mapping from nanopore direct RNA sequencing**. *Nature Communications* 2023, 14(1):1906.
3. Hu L, Liu S, Peng Y, Ge R, Su R, Senevirathne C, Harada BT, Dai Q, Wei J, Zhang L: **m6A RNA modifications are measured at single-base resolution across the mammalian transcriptome**. *Nature biotechnology* 2022, 40(8):1210-1219.
4. Linder B, Grozhik AV, Olarerin-George AO, Meydan C, Mason CE, Jaffrey SR: **Single-nucleotide-resolution mapping of m6A and m6Am throughout the transcriptome**. *Nature methods* 2015, 12(8):767-772.
